# Effects of Whole Body Therapeutic Hypothermia on Gastrointestinal Morbidity and Feeding Tolerance in Infants with Hypoxic Ischemic Encephalopathy

**DOI:** 10.1155/2014/643689

**Published:** 2014-08-25

**Authors:** Kimberly M. Thornton, Hongying Dai, Seth Septer, Joshua E. Petrikin

**Affiliations:** ^1^Department of Neonatology, Children's Mercy Hospital, 2401 Gillham Road, Kansas City, MO 64108, USA; ^2^School of Medicine, University of Missouri-Kansas City, 2401 Gillham Road, Kansas City, MO 64108, USA; ^3^Research Development and Clinical Investigation, Children's Mercy Hospital, 2401 Gillham Road, Kansas City, MO 64108, USA; ^4^Department of Gastroenterology, Children's Mercy Hospital, 2401 Gillham Road, Kansas City, MO 64108, USA

## Abstract

*Objective*. This retrospective cohort study evaluated the effects of whole body therapeutic hypothermia (WBTH) on gastrointestinal (GI) morbidity and feeding tolerance in infants with moderate-to-severe hypoxic ischemic encephalopathy (HIE). *Study Design*. Infants ≥ 35 weeks gestational age and ≥1800 grams birth weight with moderate-to-severe HIE treated from 2000 to 2012 were compared. 68 patients had documented strictly defined criteria for WBTH: 32 historical control patients did not receive WBTH (non-WBTH) and 36 cohort patients received WBTH. *Result*. More of the non-WBTH group infants never initiated enteral feeds (28% versus 6%; *P* = 0.02), never reached full enteral feeds (38% versus 6%, *P* = 0.002), and never reached full oral feeds (56% versus 19%, *P* = 0.002). Survival analyses demonstrated that the WBTH group reached full enteral feeds (median time: 11 versus 9 days; *P* = 0.02) and full oral feeds (median time: 19 versus 10 days; *P* = 0.01) sooner. The non-WBTH group had higher combined outcomes of death and gastric tube placement (47% versus 11%; *P* = 0.001) and death and gavage feeds at discharge (44% versus 11%; *P* = 0.005). *Conclusion*. WBTH may have beneficial effects on GI morbidity and feeding tolerance for infants with moderate-to-severe HIE.

## 1. Introduction

Perinatal HIE is associated with high morbidity and mortality in the neonatal period as well as long-term neurocognitive deficits. Although slightly different inclusion criteria were used among studies, multiple randomized controlled trials demonstrate that WBTH has a statistically significant improvement in neurodevelopmental disability at 18 to 24 months of [1–3] followup and 6 to 7 years of followup [4] for infants with moderate-to-severe HIE at birth. However, the effects of a perinatal hypoxic ischemic event extend beyond the brain and neurodevelopment. Decreased perfusion to the GI tract [5] and decreased motility leading to feeding intolerance [6] may follow perinatal asphyxia. Patients can present with symptoms of GI bleeding, vomiting, diarrhea, and even necrotizing enterocolitis (NEC) following HIE [7, 8]. Since WBTH acts to prevent secondary damage to the brain from ischemia and reperfusion injury that occurs following periods of perinatal anoxia, it is plausible to believe that WBTH could have similar preventative effects on the ischemic damage to the GI system.

Previous studies demonstrate conflicting results. There were some indications of hepatic dysfunction and feeding disturbances seen in subjects involved in a few of the major studies for therapeutic hypothermia, but none of those studies focused specifically on the effects to the GI system as a primary outcome. For example, the* Cool Cap* study, using selective head cooling, reported elevated liver enzymes (aspartate transaminase > 200 IU/L and alanine transaminase > 100 IU/L) in 38% of the cooled subjects versus 53% of controls (*P* = 0.02) [9]. Another selective head cooling trial, performed by Zhou et al., reported raised liver enzymes (not defined) in 35% of the cooled subjects versus 28% of the controls (*P* > 0.05) [10]. The* ICE* study reported 35% of subjects who received WBTH with hepatic dysfunction (alanine aminotransferase level > 100 U/L) versus 45% of controls (*P* > 0.05). This study also found a higher incidence of GI impairment (sloughing of the bowel, rectal bleeding, or NEC) in 4% of cooled subjects versus 2% of controls (*P* > 0.05) [2]. Another WBTH study, sponsored by the National Institute of Child Health and Human Development (NICHD), reported hepatic dysfunction (aspartate aminotransferase level > 200 IU and alanine aminotransferase level > 100 IU) in 20% of cooled subjects versus 15% of controls (significance not reported but calculated *P* > 0.05). In the cooled group, 11% of patients were discharged on gavage feeds versus 7% of controls (significance not reported but calculated *P* > 0.05) and 7% of patients were discharged with a gastric tube (GT) versus 17% of controls (significance not reported but calculated *P* > 0.05) [3]. Finally, the* TOBY* trial for WBTH reported only one case of NEC in a cooled patient and no cases of NEC in the control group (<1% versus 0%, no significance) [1].

To our knowledge, there are no published clinical trials evaluating the effects of therapeutic hypothermia for GI ischemia and reperfusion injury following HIE in humans, but several animal studies suggest a benefit. Pierro and Eaton [11] developed a rat model of intestinal ischemia-reperfusion by surgically isolating and temporarily occluding the superior mesenteric artery for 30-minute duration and then assessing signs of metabolic and histologic damage incurred to the GI tract after 60 minutes of reperfusion. They demonstrated evidence of liver energy failure, intestinal damage, and 100% mortality in the animals within 4 hours of reperfusion at normothermia. However, when the rats were kept hypothermic (32-33°C) by controlling the environmental temperature, there was a significant decrease in metabolic and histologic damage as well as 100% survival [11]. Hassoun et al. used a similar rat model with 45 minutes of induced ischemia and 6 hours of reperfusion to demonstrate decreased levels of inflammatory markers (nuclear factor kappa-B and inducible nitric oxide synthase); an increased level of a protective marker (heme oxygenase-1) and decreased histologic intestinal damage after direct hypothermia was applied to the GI tract during the ischemic period [12]. Finally, Stefanutti and colleagues [13] used the same rat model with “rescue” hypothermia applied by controlling the environmental temperature only during the reperfusion period. After 60 minutes of ischemia and 2 to 5 hours of reperfusion, hypothermia led to 100% survival and reduced inflammation, metabolic injury, and histologic damage to the GI tract [13].

We wanted to apply this evidence for WBTH use in animal models with ischemia-reperfusion injury to clinical practice. The purpose of our study was to evaluate the effects of WBTH on GI function and feeding tolerance in infants diagnosed with moderate-to-severe HIE. The primary objective was to determine the number of days to reach full enteral feeds and to reach full oral feeds in a group of infants with HIE treated with WBTH compared to a historical control group of infants with HIE prior to the routine use of WBTH. Our hypothesis was that WBTH would have a protective effect on GI function and feeding tolerance status post-moderate-to-severe HIE.

## 2. Methods

### 2.1. Study Population: Total

**Figure 1:**
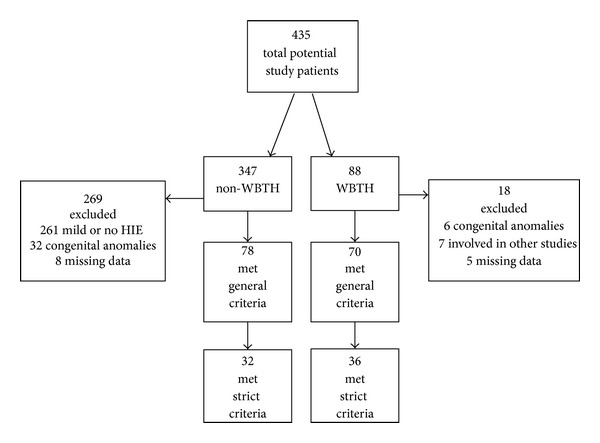
Study population.

Study approval was obtained from the Institutional Review Board of Children's Mercy Hospitals (Kansas City, MO). There were 435 total patients identified for retrospective analysis: 347 in the non-WBTH group and 88 in the WBTH group. 287 total patients were excluded from the study because they did not meet criteria for moderate-to-severe HIE, because they had a significant congenital defect at birth, because they were enrolled in another research study involving WBTH, or because there was insufficient data available in the medical record. There were 78 patients in the non-WBTH general criteria (defined below) group and 70 patients in the WBTH general criteria group. There were 32 patients in the non-WBTH strict criteria (defined below) group and 36 patients in the WBTH strict criteria group (Figure 1).

### 2.2. Study Population: Non-WBTH Group

Medical charts were reviewed for all infants ≥ 35 weeks of gestational age and ≥ 1800 grams of birth weight admitted to our institution from January 2000 to December 2008 with at least one of the following diagnoses on admission: hypoxic ischemic encephalopathy, birth asphyxia, meconium aspiration, nuchal cord, placental abruption, seizure, depression at birth, acidosis at birth, and/or shoulder dystocia. Strict criteria for the use of WBTH at our institution are based on previously published data [1–3, 9, 10, 14] and defined as a history of birth asphyxia, severe metabolic acidosis on cord blood gases or blood gas obtained within 1 hour of life (pH ≤ 7 or base deficit ≥ 16 or pH 7.01–7.15 or base deficit 10–16 and 10-minute APGAR score ≤ 5 or assisted ventilation at birth continued ≥ 10 minutes), and seizures or other evidence of moderate-to-severe encephalopathy (decreased consciousness, decreased-to-no activity, distal flexion or decerebrate posturing, hypotonia, decreased or absent reflexes, abnormal pupillary response, and abnormal breathing pattern) as documented in the medical record. Patients with adequate documentation were included in the study as historical controls and defined as the non-WBTH strict criteria group. When data for these criteria was missing, the record was reviewed further to identify other evidence of significant HIE. If patients had indirect evidence of moderate-to-severe HIE (abnormal lab values, abnormal neuroimaging, death, etc.), they were included as historical controls in the larger non-WBTH general criteria group. If the degree of HIE was considered to be mild or insufficient evidence was ultimately found to determine HIE status, the patient was excluded from the analysis. Patients with significant congenital anomalies at birth (congenital heart disease, gastroschisis, omphalocele, intestinal atresia, chromosomal trisomy, hydrocephalus, etc.) or patients involved in another study protocol using WBTH were also excluded.

### 2.3. Study Population: WBTH Group

A data set had been previously collected for quality purposes on all patients receiving WBTH from the start of standard practice use at our institution in December 2008 up to December 2012. These patients had been diagnosed with moderate-to-severe HIE based on the aforementioned criteria and completed 72 hours of WBTH at 33-34°C. In some instances, inadequate documentation existed so that all of the criteria were not found for each patient. The WBTH strict criteria group included only those patients with all criteria clearly documented, while the WBTH general criteria group included all patients who received WBTH. Again, patients with significant congenital anomalies at birth (congenital heart disease, gastroschisis, omphalocele, intestinal atresia, hydrocephalus, etc.) or patients involved in another study protocol using WBTH were excluded. Of note, patients with moribund conditions or congenital/chromosomal anomalies with poor likelihood of survival in the neonatal period were not treatment candidates as per the standard protocol for WBTH at our institution.

### 2.4. Study Design

We performed a twofold analysis of the data by comparing infants in the strict criteria group who received WBTH to those who did not and comparing infants in the general criteria group who received WBTH to those who did not. If information was missing in the medical record for the individual research parameters, the patient was excluded only from the statistical analysis of that specific parameter. Primary outcomes were day of life (DOL) enteral feeds started, DOL full enteral feeds reached, and DOL full oral feeds reached. Secondary outcomes included never started enteral feeds, never reached full enteral feeds, never reached full oral feeds, death, GT, combined death and GT, discharge with gavage feeds, combined death and discharge with gavage feeds, NEC, use of extracorporeal membrane oxygenation (ECMO), elevated liver enzymes, elevated coagulation parameters, and days to hospital discharge or death. These outcomes were chosen as objective measures of GI morbidity and feeding tolerance that could be easily determined from the electronic medical documentation. Other variables such as gastric residuals and withholding of feeds were considered for outcome measures but were not well documented in the electronic medical record and could not be used for this study.

### 2.5. Statistical Methods

Data are expressed as mean ± standard deviation (SD) for continuous outcome variables and percentage for categorical outcome variables. Characteristics of WBTH and non-WBTH patients were compared using Chi-square test, Fisher's exact test, and *t*-test. Patients who did not start or reach full enteral feeds due to death were treated as censored data. Patients who did not reach full oral feeds due to death, GT placement, or discharge with gavage feeds were also treated as censored data. Kaplan-Meier survival curves were generated for WBTH and non-WBTH patients. We then performed the log-rank test to compare survival curves between the two groups. All analyses were performed using SAS 9.2 (Cary, NC) and SPSS 20. Statistical significance was claimed at 95% confidence level (*P* < 0.05).

## 3. Results

### 3.1. Comparison of Demographic Data

Overall, there were more males (59% of total study population) than females and more Caesarean sections performed (66% of total study population) than vaginal deliveries for infants with moderate-to-severe HIE. There were slight differences in birth weight, gestational age, and maternal age between the groups, but we do not feel that these differences affected our results. The degree of HIE, defined by the 5-minute APGAR score, was similar for the general criteria group, but the strict WBTH group had lower APGAR scores at 5 minutes when compared with the non-WBTH group (2.2 ± 8 versus 4.1 ± 1.8, *P* < 0.001). However, the mean score was below 5 at 5 minutes for both groups, suggesting that the groups had comparable degrees of depression at birth (Table 1).

### 3.2. Comparison between WBTH (*n* = 36) and Non-WBTH (*n* = 32) Using Strict Criteria

**Figure 2:**
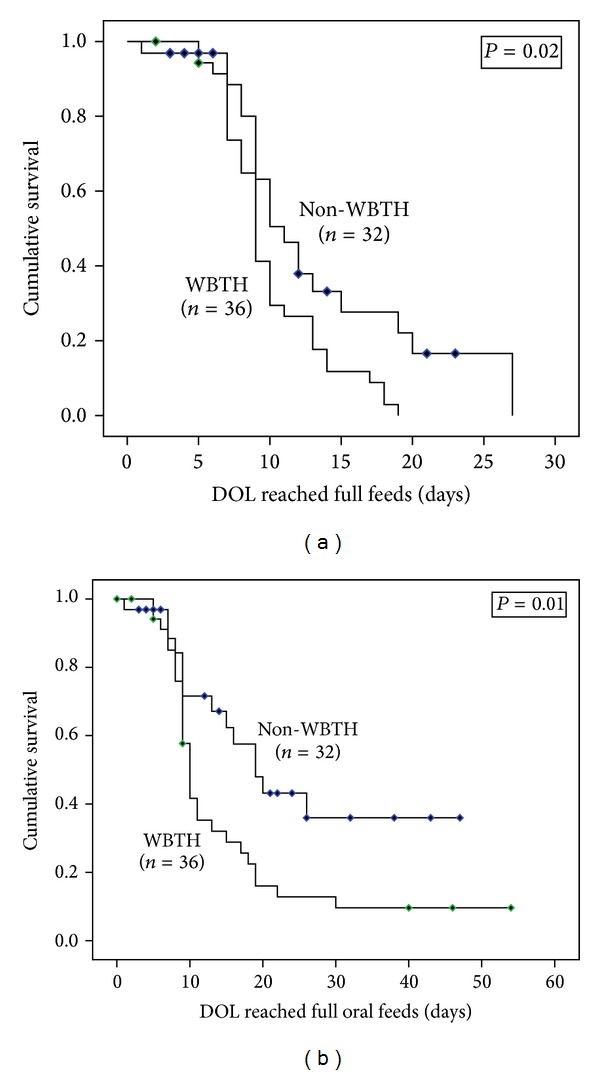
Survival curves for subjects with strict criteria^∗^. ^∗^Censored subjects are labeled.

The non-WBTH group had more infants who never started on enteral feeds due to death (28% versus 6%, *P* = 0.02), more infants who never reached full enteral feeds due to death (38% versus 6%, *P* = 0.002), and more infants who never reached full oral feeds due to death, GT placement, or discharge with gavage feeds (56% versus 19%, *P* = 0.002) as compared to the WBTH group. The non-WBTH group has a higher mortality rate than the WBTH group (32% versus 6%, OR (95% CI): 7.9 (1.6–39.5), *P* = 0.009). The non-WBTH group had a higher rate of combined death and GT placement (46.9% versus 11.1%, *P* = 0.001) and combined death and gavage feeds at discharge (43.8 versus 11.1, *P* = 0.005). Survival analysis was performed to take the censoring into account. The Kaplan Meier curves demonstrated that infants in the WBTH group reached full enteral feeds (median time: 11 versus 9 days, *P* = 0.02) and full oral feeds (median time: 19 versus 10 days, *P* = 0.01) sooner than those in the non-WBTH group (Figure 2). There were only 3 cases of NEC, but they were all in the non-WBTH group (9% versus 0%, *P* = 0.1) There was no difference in elevation of liver enzymes (63% versus 63.9%, *P* = 0.94) or abnormal coagulation factors (91.3% versus 100%, *P* = 0.15) between groups (Table 2).

### 3.3. Comparison between WBTH (*n* = 70) and Non-WBTH (*n* = 78) Using General Criteria

There was no statistical significance between groups for primary outcomes, mortality, or GI morbidity measures. There were few cases of NEC, and there was no statistical significance of occurrence of NEC between groups (5% versus 3%, *P* = 0.68). However, there were an increased number of infants who never reached full oral feeds due to death, GT placement, or discharge with gavage feeds in the non-WBTH group versus the WBTH group (42% versus 26%, *P* = 0.03). Also, infants who received WBTH had elevated coagulation parameters (prothrombin time > 15.6 seconds and partial thromboplastin time > 41.5 seconds) (80% versus 100%, *P* = 0.0001) more often than those not treated with WBTH, but there was no difference in the number of infants with elevated liver enzymes between groups (54.6% versus 62.9%, *P* = 0.33) (Table 3).

## 4. Discussion

Our analysis suggests that infants treated with WBTH for moderate-to-severe HIE may have improved feeding tolerance, GI morbidity, and overall mortality compared with those not treated with WBTH. Infants treated with WBTH strict criteria group reached full enteral feeds and full oral feeds sooner than those not treated. The WBTH strict criteria group had a lower overall mortality as well as combined GI morbidity (defined as GT placement or gavage feeds at discharge) and mortality.

Our results show that the majority of subjects had elevated liver enzymes and elevated coagulation parameters, expected sequelae of HIE [15, 16]. Other studies have reported that these effects were only temporary with improvement in liver enzymes and coagulation parameters over time, and our results were consistent (data not shown). There was, however, no significant difference in the number of infants with elevated liver enzymes between the WBTH and non-WBTH groups in our study.

Interestingly, there was no difference in the DOL feeds initiated between groups in either analysis. This retrospective study covered a wide time frame during which many changes occurred in medical practice, including the standard use of WBTH for moderate-to-severe HIE and the use of standardized feeding protocols. The feeding protocols are primarily used for the initiation and advancement of feeds in preterm infants. There is no current standardized practice for initiating feeds in late-preterm and term infants with HIE at our institution. However, we expected to see a difference in practice for initiation of feeds over the twelve-year timeframe of our study, believing that many providers would feel more comfortable starting feeds sooner in the group of infants treated with WBTH. Since this was not the case, our results are actually strengthened by the fact that both groups were fed similarly.

Limitations of the study include the small sample size and retrospective analysis of data. A historical cohort study was performed because therapeutic hypothermia has been accepted as the standard of care for infants with moderate-to-severe HIE; a prospective analysis or randomized controlled trial is not an ethical option to evaluate the effects of WBTH on GI morbidity and feeding following a perinatal hypoxic event. We estimate that fewer infants with HIE were referred to our tertiary care institution prior to use of WBTH due to the lack of therapeutic options, further limiting the number of patients available for analysis. The study was also limited by the quality of documentation in the medical record. This was the reason for dividing the groups into strict and general criteria based on the level of documentation available. When there was a lack of adequate information, the research team either determined an infant that met criteria for moderate-to-severe HIE based on other findings in the medical record (such as multiple abnormal lab values or even death) or decided to exclude the infant from the study if insufficient evidence was found. Although the 5 minute APGAR score was lower in the non-WBTH strict group versus the WBTH strict group, both groups had a mean score <5 at 5 minutes of life to suggest a considerable level of depression after birth in each one. The lower mortality seen specifically in the WBTH strict criteria group may arguably be explained by the fact that these infants were already a subgroup of infants with more significant illness. However, the lower mortality combined with more significant results seen in the groups of infants with clear evidence of moderate-to-severe HIE by a more strict definition validates the strict use of cooling criteria used by our institution.

It should be noted that a requirement for GT placement or gavage feeds at discharge or the number of days to reach full oral feeds could all be reflections of oral skills. Those differences seen in our study could arguably be similar to the known improvements in neurologic function seen with WBTH. However, advancement of enteral feeds is a more specific measure of GI motility and the decreased number of days to reach full enteral feeds alone suggests a nonneural benefit to WBTH.

Future implications for study may include use of therapeutic hypothermia for other causes of gastrointestinal ischemia-reperfusion injury such as NEC. NEC is a unique form of gastrointestinal ischemia that occurs most often in preterm infants. Its exact etiology is not fully elucidated and felt to be multifactorial. In 2010, Hall et al. [17] published a small randomized prospective pilot study establishing the safety of using WBTH in preterm infants with advanced stage surgical NEC. The authors of the study plan to complete a larger multicenter randomized controlled trial evaluating the use of therapeutic hypothermia to decrease the amount of GI ischemic injury and surgical intervention required for NEC. Although our study was not powered for the incidence of NEC, we see interesting trends in the small numbers of patients with NEC and moderate-to-severe HIE. Our study strongly supports the need for further research in this area. Next steps could include a larger, multicentered cohort study evaluating the incidence of NEC in infants with HIE before and after the use of WBTH.

In conclusion, WBTH use for moderate-to-severe HIE may have beneficial effects to the newborn that extend beyond neurocognitive and neurodevelopmental outcomes. Our findings suggest that WBTH improves GI morbidity and feeding tolerance for infants with moderate-to-severe HIE when strict criteria for cooling are applied.

## Figures and Tables

**Table 1:** Demographics for all subjects.

Variable	WBTH	Non-WBTH	*P* value
General criteria group	(*n* = 70)	(*n* = 78)	

Gender (% male)	44 (63%)	44 (56%)	0.50
Birth weight (grams)	3447.1 ± 524.2	3262.0 ± 570.3	0.04∗
Gestational age (weeks)	39.0 ± 1.5	38.4 ± 1.9	0.02∗
Maternal age (years)	25.4 ± 6.3	29.8 ± 15.6	0.03∗
Cesarean delivery	45 (64%)	52 (67%)	0.86
5-minute APGAR score	3.0 ± 2.1	3.3 ± 2.0	0.29

Strict criteria group	(*n* = 36)	(*n* = 32)	

Gender (% male)	18 (50%)	18 (56%)	0.63
Birth weight (grams)	3510 ± 523.7	3120.1 ± 568.6	<0.01∗
Gestational age (weeks)	39.2 ± 1.5	38.3 ± 1.7	0.02∗
Maternal age (years)	25.4 ± 6.9	27.9 ± 7.6	0.17
Cesarean delivery	21 (58%)	23 (72%)	0.31
5-minute APGAR score	4.1 ± 1.8	2.2 ± 1.8	<0.001∗

∗Significance defined by *P* < 0.05.

**Table 2:** Primary and secondary outcome variables for strict criteria group.

Variable	WBTH (*n* = 36)	Non-WBTH (*n* = 32)	*P* value
Primary outcomes (mean ± SD)			
DOL enteral feeds started	5.6 ± 2	6 ± 3	0.12
DOL full enteral feeds reached	10.1 ± 3.7	11.3 ± 5.6	0.02∗
DOL full oral feeds reached	11.5 ± 5.6	12.7 ± 6.7	0.01∗

Secondary outcomes (%)			
Never started enteral feeds	5.6	28.1	0.02∗
Never reached full enteral feeds	5.6	37.5	0.002∗
Never reached full oral feeds	19.4	56.3	0.002∗
Death	5.7	32.3	0.009∗
GT	5.6	15.6	0.24
Combined death and GT	11.1	46.9	0.001∗
Discharged with gavage feeds	5.6	9.4	0.66
Combined death and discharged with gavage feeds	11.1	43.8	0.005∗
NEC^+^	0	9.4	0.1
ECMO	8.3	0	0.24
Elevated liver enzymes^†^	63.9	63	0.94
Elevated coagulation parameters^∞^	100	91.3	0.15
Days to hospital discharge or death	16.9 ± 11.1	16.9 ± 12	1

∗Significance defined by *P* < 0.05.

^+^Grossly bloody stool (Bell's stage IIA or greater).

^†^Aspartate aminotransferase level >200 IU and alanine aminotransferase level >100 IU.

^∞^Prothrombin time >15.6 seconds and partial thromboplastin time >41.5 seconds.

**Table 3:** Primary and secondary outcome variables for general criteria group.

Variable	WBTH (*n* = 70)	Non-WBTH (*n* = 78)	*P* value
Primary outcomes (mean ± SD)			
DOL enteral feeds started	5.4 ± 2.2	5.8 ± 2.6	0.39
DOL full enteral feeds reached	9.9 ± 4.3	10.4 ± 6.1	0.12
DOL full oral feeds reached	11.4 ± 6.2	11.4 ± 6.9	0.13

Secondary outcomes (%)			
Never started enteral feeds	12.9	21.8	0.15
Never reached full enteral feeds	12.9	25.6	0.05
Never reached full oral feeds	25.7	42.3	0.03∗
Death	15.9	23.4	0.26
GT	10	12.8	0.29
Combined death and GT	26	36.4	0.21
Discharged with gavage feeds	2.9	7.7	0.28
Combined death and discharged with gavage feeds	17.4	31.2	0.06
NEC^+^	2.9	5.1	0.68
ECMO	5.7	1.3	0.19
Elevated liver enzymes^†^	62.9	54.6	0.33
Elevated coagulation parameters^∞^	100	80.4	0.0001∗
Days to hospital discharge or death	15.8 ± 10.2	16.2 ± 10.9	0.83

∗Significance defined by *P* < 0.05.

^+^Grossly bloody stool (Bell's stage IIA or greater).

^†^Aspartate aminotransferase level >200 IU and alanine aminotransferase level >100 IU.

^∞^Prothrombin time >15.6 seconds and partial thromboplastin time >41.5 seconds.
